# Robust Alpha Spending for Unstable Data

**DOI:** 10.1002/sim.70760

**Published:** 2026-10-09

**Authors:** Ivair R. Silva, Judith C. Maro

**Affiliations:** ^1^ Department of Population Medicine Harvard Pilgrim Health Care Institute Boston Massachusetts USA; ^2^ Computer Department Federal University of Ouro Preto Ouro Preto Brazil; ^3^ Department of Population Medicine Harvard Medical School Boston Massachusetts USA

**Keywords:** adverse events, expected time to signal, post‐market vaccine safety surveillance, relative risk, sequential test

## Abstract

Sequential hypothesis testing has been used in randomized clinical trials and in monitoring adverse events after vaccination. Typically, the presumption is that all data that have been previously used in the sequential analysis remain the same, and only new data are added. We call this the “anchoring” assumption. In prelicensing randomized trials, enrollment goals are set based on these sequential hypothesis tests so that a trial may stop if a drug proves either too hazardous or, conversely, so beneficial that it would be unethical to deny the treatment to others. However, in monitoring adverse events after vaccination, data are mostly “secondary use,” or real‐world data. That is, these data are not gathered for research, but are clinical data that have been repurposed for observational vaccine safety surveillance. As such, the counts of exposed persons and adverse events may be subject to revisions over time. These data changes directly challenge the anchoring assumption upon which most sequential hypothesis testing depends. If these data changes are discovered prior to subsequent hypothesis tests, it is unclear how to account for these changes in subsequent tests. We introduce a solution: “robust alpha spending” to protect overall type I error probability, statistical power, and surveillance time from low‐level data instability present in secondary use data sources used for adverse event monitoring.

## 1 Introduction

When a new drug or vaccine is commercialized, it is important to monitor adverse events subsequent to use. A robust monitoring scheme often includes multiple epidemiological designs coupled with a sequential hypothesis testing approach to quickly and rigorously detect any increased risks [1]. Typically, multiple sequential hypothesis tests are applied cumulatively to non‐randomized data over time, enabling prospective surveillance [2]. Such methods were pioneered by the U.S. Centers for Disease Control and Prevention's Vaccine Safety Datalink using data from electronic health records and are also called “rapid cycle analysis” [3, 4, 5].

Sequential test methods are based on comparing a test statistic with a signaling threshold. A number of sequential test methods have been utilized in clinical trials [6, 7], such as Pocock's Score Statistic [8], O'Brien & Fleming [9], and Wang‐Tsiatis' test [10]. For postmarket medical product safety surveillance of adverse events, a common method is the maximized sequential probability ratio test (MaxSPRT) [11]. Most of these methods employ alpha spending functions that prespecify how type I error probability will be spent over a series of sequential hypothesis tests.

These methods make implicit assumptions around data stability when planning alpha spending functions, what we refer to as the “anchoring assumption.” That is, it is assumed that data used in a prior hypothesis test are unchanging and can be concatenated with new data for subsequent hypothesis tests. Similarly, alpha that has previously been “spent” is deducted from a total type I error allowance to determine remaining alpha available for spending in new hypothesis tests. In a clinical trial environment that relies on primary data collection from research subjects, this assumption is never in jeopardy. However, this assumption can fail in postmarket observational safety surveillance that uses data collected in routine clinical care and repurposes it for surveillance [12]. A simple example is when an initial diagnosis evolves over time such that first recorded diagnoses are not an accurate depiction of the patient's condition. Because the data sources used are often administrative claims databases, other sources of changes may be based on audits or other investigations that find that diagnoses were inappropriately given. Changes in the data may lead to serious biases in statistical inferences, such as inflation in the type I error probability or loss of statistical power.

To illustrate, consider a two‐stage sequential test for a self‐controlled design where there is a risk window specified after vaccination (i.e., days 1–28) and a control window that occurs after the risk window (i.e., days 29–84). For time period 1, suppose that $M_{1}$ subjects are exposed to a vaccine and contribute observed time to both the risk window and the control window. If $n_{1}$ adverse events are observed, then the number of adverse events that are expected to be in the risk window, say $X_{1}$, can be modeled through a binomial distribution with parameters $n_{1}$ and $p_{1}(R)$, where $p_{1}(R)$ is given by:

(1) $$p_{1}(R)=\frac{1}{1+z_{1}/R},$$

where $z_{1}$ is the ratio of contributed time in the control window to the risk window when data are analyzed in the first sequential hypothesis test, and R is the relative risk of an adverse event during the risk window. A common format of the hypotheses to test the safety of the vaccine in this context is

$$\begin{array}{ll} H_{0} & :R\leq 1,\\ H_{1} & :R>1, \end{array}$$

For the test criteria, the null will be rejected in time t if $Y_{t}\geq cv_{t}$, t=1,2, where $Y_{1}=X_{1}$, $Y_{2}=X_{1}+X_{2}$, and $cv_{t}$ is the signaling threshold, or critical value, in time t which is operationalized as the number of events needed in the risk window to reject the null hypothesis at time t. Further suppose that an overall alpha level of 0.05 is planned to be evenly split among the two sequential hypothesis tests to be performed, that is, up to 0.025 can be spent in test 1. For example, if $z_{1}=2$, thus $p_{1}(1)={\left(1+2\right)}^{-1}=0.3333$, and $n_{1}=4$, then the solution is $cv_{1}=4$ events, which generates an alpha expenditure of $P(Y_{1}\geq 4;R=1)\approx 0.012346$. That is, among the 4 events that accrued in time period 1, one third of them are expected to occur in the risk window and the remainder in the control window. All 4 events would have to have been observed during the risk window to reject the null hypothesis. If the null hypothesis is not rejected in the first test, then surveillance continues and the analyst will wait for the second test. Now, suppose that $n_{2}=3$ events arrived in time 2. If $z_{2}=2$, and thus $p_{2}(1)=0.3333$, then $cv_{2}$ is the largest value of c such that:

$$P(Y_{1}\geq 4;R=1)+P(Y_{1}<4,\;Y_{2}\geq c;R=1)\leq 0.05,$$

which gives $cv_{2}=5$ events. That is, among the total of 7 adverse events, 5 of those would have to have been observed in the risk window to reject the null hypothesis. With these circumstances, after running the calculations above, the overall alpha spending after two hypothesis tests is approximately equal to 0.0489, which is within the allowable type I error probability.

Now, say all four events in time period 1 were first recorded in the risk window, and the null hypothesis had been rejected. If one of those events was latently discovered to be in the control window (i.e., resulting in a 3/1 split instead of a 4/0 split between the risk and control windows, respectively), then the amount of alpha that would have been required to reject the null hypothesis with just 3 events is $P(Y_{1}\geq 3;R=1)\approx 0.1111$, which, besides being much higher than the planned alpha spending (i.e., up to 0.025), is over twice higher than the overall 0.05 level.

It is also possible that data move in the reverse direction, although this is a less likely scenario in the self‐controlled design example. Nonetheless, it could happen in a number of other epidemiological designs if exposure is misclassified. If there are less control window cases and more risk window cases due to misclassification, the statistical power by the end of the sequential analysis would be smaller than the target power presumed and planned for in the sequential analysis plan. Similar harmful effects will occur if it is discovered that an adverse event was not a true event at all and was removed from the study before the upcoming test.

The conventional practice for choosing an alpha spending plan in postmarket vaccine safety surveillance is to set the overall type I error probability and then specify a target statistical power, while minimizing expected time‐to‐signal [13]. However, as shown in the illustrative example above, data instability may thwart either false positive control or statistical power and produce incorrect statistical inferences. In practice, many sequential monitoring studies avoid looking at the same data twice and may be unaware of data instability, or they may a priori specify that the data will be “frozen” or “locked” at the time it was analyzed even if it is shown later to be incorrect [12, 14]. Other approaches have simply restarted alpha spending plans as though alpha were never spent. None of these solutions are formal or satisfactory. Therefore, to the best of our knowledge, there are no available methods to handle unstable data in the sequential analysis of adverse events.

In the present article, we introduce the concept of “robust alpha spending” for managing the effects of unstable data. The rationale is to plan the spend of the type I error probability in a way that the overall target type I error allowance and the target power are assured by the end of the analysis. Although the impact of unstable data will be unknown at the start of the prospective study, under reasonable practical conditions dictated by the analyst, statistical performance measures are kept controlled by allowing for additional sources of variance (i.e., data instability). Along with the toy example presented here, we have previously illustrated the problem with real world data [12], but did not have a solution proposed at that time.

In Section 2, we define robust alpha spending for binary/binomial, Poisson and conditional Poisson data used in sequential hypothesis testing to support postmarket safety surveillance. Briefly, the choice of whether to use a binary/binomial or a Poisson/conditional Poisson statistical model is dictated by study design [13]. The former is generally used with a cohort design and a concurrent comparator group, or when there is a self‐controlled design. The latter is generally used when there is a cohort design with a historical comparator group. The conditional Poisson variant is preferred when the historical comparator group is less than five times as large as the planned surveillance group [15] because it accounts for additional uncertainty in the background rate. Gandhi et al.'s literature review of recently completed sequential hypothesis testing studies for vaccines found a supermajority used historical comparator groups, particularly in pandemic‐related studies [1]. However, the use of multiple study designs with multiple comparator groups has also been common practice and necessitates the use of multiple sequential hypothesis testing models [16, 17, 18].

The robust alpha spending approach was implemented in the R *Sequential* package [19], which was used to perform the simulation study described in Section 3. Section 4 illustrates the use of the proposed method with real and simulated data. The real data was extracted from [12]. We conclude in Section 5.

## 2 Robust Alpha Spending

We say that a target alpha spending plan is robust to a set C of possible data changes if, for limited deviations from C: (i) the actual overall type I error probability is controlled under the nominal overall significance level, α, and (ii) the target statistical power of interest is greater than or equal to the target power, γ. We return to these two constraints as we introduce the formal construction of the robust alpha spending definition separately for binary/binomial, Poisson, and conditional Poisson data, respectively. In each case, the parameter of interest is the relative risk, R, and the format of the hypothesis pair is:

$$\begin{array}{ll} H_{0} & :R\leq r_{0},\\ H_{1} & :R>r_{0}, \end{array}$$

where $r_{0}$ is a test margin that is typically set to 1 but may be greater if desired.

Overall, in any sequential hypothesis testing approach, the statistical power available at the end of the analysis depends on how increments of data arrive for new hypothesis tests. With a fixed type I error constraint, the smallest cumulative statistical power occurs when each adverse event has its own hypothesis test (i.e., truly continuous sequential surveillance) [20]. This data arrival pattern may be possible with truly rare conditions like Guillain‐Barre Syndrome. Because our robust alpha spending construction guarantees a minimum target power, we make the most conservative assumption regarding data arrival (i.e., individual hypothesis tests). Any irregular data arrival—where groups of adverse events arrive together for a hypothesis test—will improve statistical power above the minimum.

Although the direction and magnitude of unstable data changes are unknown at the time of analysis, we consider 4 specific cases that will affect sequential hypothesis testing

$s_{n}^{+}$: the cumulative number of events that are later removed from the analysis because they were latently discovered not to be true events;

$s_{n}^{-}$: the cumulative number of actual events that were missed at the time of the sequential hypothesis test;

$e_{n}^{+}$: the cumulative number of events wrongly classified as exposed when they were actually unexposed;

$e_{n}^{-}$: the cumulative number of events wrongly classified as unexposed when they were actually exposed.

### 2.1 Binary and Binomial Data

As mentioned above, the Binomial MaxSPRT can be used with self‐controlled designs or cohort designs including matched, stratified, or weighted designs [13]. In self‐controlled designs, the matching ratio is typically dependent on the length of the risk and control windows. In cohort designs, the ratio of the time contributed by the treatment group relative to the overall study population at the time of the event can be operationalized as a matching ratio.

Let $X_{1},X_{2},\ldots ,$ denote a sequence of independent Bernoulli random variables, where the success probability associated to $X_{j}$ is equal to $p_{j}(R)$, given by

(2) $$p_{j}(R)=\frac{1}{1+z_{j}/R},$$

where $z_{j}$ is the matching ratio associated to $X_{j}$, j=1,2,….

For a signaling threshold given in the scale of a test statistic like, for example, Pocock, O'Brien‐Fleming, MaxSPRT, and Wang‐Tsiatis, the critical values can be rewritten in the scale of observed events: $Y_{n}=\sum_{j=1}^{n}X_{j}$, [13]. Let ${\text{cv}}_{n}=(cv_{1},\;cv_{2},\;\ldots ,\;cv_{n})$ denote the vector of critical values, with n=1,2,…,N, where N is the maximum length of surveillance (in the scale of observed events) to stop the analysis without rejecting the null hypothesis. Therefore, the regular test decision is based on rejecting the null hypothesis when $X_{n}\geq cv_{n}$ for some 1≤n≤N.

Let vectors with the time‐by‐time information related to the four counting errors described above be denoted by

$$\begin{array}{ll} & s_{n}^{+}=(s_{1}^{+},\;s_{2}^{+},\ldots ,\;s_{n}^{+}),\\ & s_{n}^{-}=(s_{1}^{-},\;s_{2}^{-},\ldots ,\;s_{n}^{-}),\\ & e_{n}^{+}=(e_{1}^{+},\;e_{2}^{+},\ldots ,\;e_{n}^{+}),\\ & e_{n}^{-}=(e_{1}^{-},\;e_{2}^{-},\ldots ,\;e_{n}^{-}). \end{array}$$

If the time index of the lth hypothesis test is n, then the actual number of events at look l is $\eta_{l}=n-s_{n}^{+}+s_{n}^{-}$, and the associated actual critical value is equal to $CV_{l}=cv_{n}-e_{n}^{+}+e_{n}^{-}$. Therefore, the actual implicit power function up to time n is given by

(3) $$\begin{array}{ll} A_{n}(R,{\text{cv}}_{n},s_{n}^{+},s_{n}^{-},e_{n}^{+},e_{n}^{-}) & =P(\text{rej.}\;H_{0}\;\text{up to}\;n;\;R,{\text{cv}}_{n},s_{n}^{+},s_{n}^{-},e_{n}^{+},e_{n}^{-})\\ & =P(Y_{\eta_{1}}\geq CV_{1};\;R)+\\ & +P(Y_{\eta_{1}}<CV_{1},\;Y_{\eta_{2}}\geq CV_{2};\;R)+\cdots +\\ & +P\left(\cap_{l=1}^{k-1}Y_{\eta_{l}}<CV_{l},\;Y_{\eta_{k}}\geq CV_{k};\;R\right), \end{array}$$

where k is the number of hypothesis tests. Note that the power function in (3) is the actual alpha spending when evaluated for $R=r_{0}$.

In practice, the numbers $s_{n}^{+}$, $s_{n}^{-}$, $e_{n}^{+}$ and $e_{n}^{-}$ are expected to have an upper limit on the size of the deviation at each look at the data, such as, for example, 10% or less. Thus, assuming that the analyst is able to establish such a safety margin of changes in the data, consider the user‐specified positive integers $S_{n}^{+}$, $S_{n}^{-}$, $E_{n}^{+}$ and $E_{n}^{-}$, representing the maximum values for the numbers $s_{n}^{+}$, $s_{n}^{-}$, $e_{n}^{+}$ and $e_{n}^{-}$.

Definition 2.1 (Robust alpha spending for binary data) For fixed counting error margins $S_{n}^{+}$, $S_{n}^{-}$, $E_{n}^{+}$, $E_{n}^{-}$, nominal significance level, α, desired statistical power, γ, and a target alpha spending, F(n), with a corresponding critical value vector, ${\text{cv}}_{n}$, F(n) is *robust to unstable data* conditioned on $S_{n}^{+}$, $S_{n}^{-}$, $E_{n}^{+}$ and $E_{n}^{-}$ if:

$$\begin{array}{ll} \;(i) & \;\max\left(A_{n}(R=r_{0},{\text{cv}}_{n},S_{n}^{+},\text{0},E_{n}^{+},\text{0}),\;\right.\\ & \left.\;\;A_{n}(R=r_{0},{\text{cv}}_{n},\text{0},S_{n}^{-},E_{n}^{+},\text{0})\right)\leq F(n),\\ \left(ii\right) & \;\min\left(A_{N}(R=r_{1},{\text{cv}}_{N},S_{N}^{+},\text{0},\text{0},E_{N}^{-}),\;\right.\\ & \left.\;\;A_{N}(R=r_{1},{\text{cv}}_{N},\text{0},S_{N}^{-},\text{0},E_{N}^{-})\right)\geq \gamma , \end{array}$$

where N is the maximum length of surveillance, $r_{1}$ is the target relative risk for the desired power, and 0 is the n‐dimensional vector of zeros. Condition (i) is consistent with our first definitional constraint: that Type I error is maintained below a maximum allowance. Condition (ii) reflects our second constraint: that target power by the end of the analysis will still meet a guaranteed minimum threshold.

One final concern specific to the calculation of statistical power for binomial data is matching ratios, which are unknown in advance in some epidemiologic designs. For these circumstances, one must establish an upper bound during planning, say $\bar{z}$. The $\bar{z}$, or fixed z, is a dependency when calculating the maximum length of surveillance to ensure a minimum target power in part (ii) of Definition 2.1.

### 2.2 Poisson Data

The Poisson MaxSPRT is based on comparing surveillance data from a cohort to a reference set of data [11]. The adverse event rate in the reference set is presumed to be known without error. Because the reference set of data is entirely collected before the surveillance data begins, there is no possibility of crossover errors (data assigned to an exposed group is unexposed or vice versa).

Let $C_{t}∼Poisson(R\mu_{t})$ represent the cumulative number of adverse events observed until time t, where t is a continuous time index, and $\mu_{t}$ is a known baseline rate. Also, let $t_{1},\;t_{2},\ldots$ denote the sequence of testing times. This way, for a given alpha spending plan, F(t), the vector with n critical values, ${\text{cv}}_{n}=(cv_{t_{1}},\;cv_{t_{2}},\;\ldots ,\;cv_{t_{n}})$, can be calculated numerically by running the absorbing states of a Poisson Markov chain, [21].

The number of actual events that are missed in the previous look at the data, when identified, can be added to the data of the current test, as this will not inflate the final significance level. Therefore, the data instability to be managed in the course of the surveillance is the number of non‐real events that are added to the study during a sequential hypothesis test but later drop out. Define:

$s_{n}^{+}$: the cumulative number of events added to the observed data incorrectly until the nth test.

For the nth test, the actual number of events is $C_{t}-s_{n}^{+}$. Therefore, the actual critical value in the scale of $C_{t}$ is equal to $CV_{t_{n}}=cv_{t_{n}}-s_{n}^{+}$. Thus, the actual implicit power function up to time t is given by

(4) $$\begin{array}{ll} A_{t}(R,{\text{cv}}_{n},s_{t}^{+}) & =P(\text{reject}\;H_{0}\;\text{until the}\;n\text{th test};\;R,{\text{cv}}_{n},s_{n}^{+})\\ & =P(C_{t_{1}}\geq CV_{t_{1}};\;R)+\\ & +P(C_{t_{1}}<CV_{t_{1}},\;C_{t_{2}}\geq CV_{t_{2}};\;R)+\cdots +\\ & +P\left(\cap_{l=1}^{n-1}C_{t_{l}}<CV_{t_{l}},\;C_{t_{n}}\geq CV_{t_{n}};\;R\right). \end{array}$$

Definition 2.2 (Robust alpha spending for Poisson data) For a fixed counting error margin $S_{t}^{+}$, nominal significance level, α, desired statistical power, γ, and a target alpha spending, F(t), with a corresponding critical value vector, ${\text{cv}}_{t}$, F(t) is *robust to unstable data* conditioned on $S_{t}^{+}$ if:

$$\begin{array}{ll} \left(i\right) & \;A_{t}(R=r_{0},{\text{cv}}_{n},S_{t}^{+})\leq F(t),\;\text{for each}\;t\leq T,\\ \left(ii\right) & \;A_{T}(R=r_{1},{\text{cv}}_{N},\text{0})\geq \gamma , \end{array}$$

where N is the maximum length of surveillance given in the scale of the events associated to the maximum surveillance time T, and $r_{1}$ is the target relative risk for the desired power.

### 2.3 Conditional Poisson Data

The conditional MaxSPRT, CMaxSPRT, was introduced by [15] for cohort designs with a reference group, similar to when one would use a Poisson MaxSPRT. However, a CMaxSPRT is appropriate when the baseline reference rate $m_{t}$ is not considered deterministic (as it is in the Poisson MaxSPRT). The CMaxSPRT is based on comparing historical and surveillance information for testing the hypothesis that the relative risk of the monitored data is equal to that of the historical reference data.

Following the same notation by [15], use V and c to denote the person‐time and the number of events from the historical data, respectively. For the surveillance data, let $P_{k}$ denote the person‐time up to the arrival of the kth event. From the Poisson process assumption, it holds that V is the sum of c independent exponential random variables with expectation 1/λ, plus a random person‐time residual, U, and $P_{k}$ follows a Gamma distribution with shape k and scale 1/(R×λ), where λ is the unknown common rate between the two periods.

As demonstrated by [22], the calculation of critical values for the CMaxSPRT, after observing the kth event, only depends on the joint distribution of the ratios:

(5) $$\tau_{j}=P_{j}/V,\;j=1,\;2,\ldots ,\;k.$$

Again, the stochastic process can be reversed to establish the monitoring on the scale of the events. This way, for fixed $\tau_{j}$, the exact distribution of the number of events, say $\kappa_{\tau_{j}}$, is obtained analytically, [22].

As in Poisson data, there are no crossover data errors, and the events missed in the previous tests, if identified, are added to the data of the next test. However, the number of events added that are later dropped must be considered in the robust alpha spending. Define:

$s_{j}^{+}$: the cumulative number of events added to the observed data by mistake until the jth test.

For the jth test, the real number of events is $\kappa_{\tau_{j}}-s_{j}^{+}$. Hence, the actual critical value in the scale of $\kappa_{\tau_{j}}$ is equal to $CV_{\tau_{j}}=cv_{\tau_{j}}-s_{j}^{+}$. The implicit power function up to time $\tau_{j}$ is given by

(6) $$\begin{array}{ll} A_{\tau_{j}}(R,{\text{cv}}_{j},s_{j}^{+}) & =P(\text{reject}\;H_{0}\;\text{until the}\;j\text{th test};\;R,{\text{cv}}_{j},s_{j}^{+})\\ & =P(\kappa_{\tau_{1}}\geq CV_{\tau_{1}};\;R)+\\ & \;+P(\kappa_{\tau_{1}}<CV_{\tau_{1}},\;\kappa_{\tau_{2}}\geq CV_{\tau_{2}};\;R)+\cdots +\\ & \;+P\left(\cap_{l=1}^{j-1}\kappa_{\tau_{l}}<CV_{\tau_{l}},\;\kappa_{\tau_{j}}\geq CV_{\tau_{j}};\;R\right), \end{array}$$

where ${\text{cv}}_{j}=(cv_{\tau_{1}},\;cv_{\tau_{2}},\ldots ,\;cv_{\tau_{j}})$.

Definition 2.3 (Robust alpha spending for Conditional Poisson data) For a fixed counting error margin $S_{j}^{+}$, nominal significance level, α, desired statistical power, γ, and a target alpha spending, F(j), with a corresponding critical value vector, ${\text{cv}}_{j}$, F(j) is *robust to unstable data* conditioned on $S_{j}^{+}$ if:

$$\begin{array}{ll} \left(i\right) & \;A_{\tau_{j}}(R=r_{0},{\text{cv}}_{j},S_{j}^{+})\leq F(j),\;j=1,\;2,\ldots ,\;K,\\ \left(ii\right) & \;A_{\tau_{K}}(R=r_{1},{\text{cv}}_{K},\text{0})\geq \gamma , \end{array}$$

where K is the maximum length of surveillance given in the scale of the events, and $r_{1}$ is the target relative risk for the desired power.

## 3 Simulation Study

Here, we use Monte Carlo simulation to demonstrate the performance of the robust alpha spending for managing unstable data. Following Definitions 2.1, 2.2, and 2.3, the proposed method should be able to ensure the overall type I error allowance and target power when the amount of data changing in time is not greater than the bounds prespecified by the user in items (i) and (ii). The three data models were simulated for α=0.05 under $r_{0}=1$, and power of γ=0.9 under $r_{2}=2$. Each scenario was run for 1000 Monte Carlo replications using R=1 to estimate Type I error control, and R=2 to study the statistical performance achieved under the alternative hypothesis. The calculations were executed through the R *Sequential* package.

### 3.1 Binary/Binomial Data

For this simulation study, binomial data was generated using a fixed matching ratio z=1, which is equivalent to a Bernoulli probability under $H_{0}$, $p_{j}(1)$, equal to 0.5. Besides being a common ratio used in real applications [1, 2, 12, 14], the choice z=1 represents a balance between favorable scenarios with a small $p_{j}(1)$ and unfavorable scenarios with a large $p_{j}(1)$ for the robust approach. Large values of $p_{j}(1)$ would rarely generate controls wrongly classified as cases because of the high frequency of the cases, leading one to intuit that there is less practical appeal in using a robust alpha spending approach. Conversely, a small $p_{j}(1)$ value has a higher probability that controls would be wrongly classified as cases and deserves more serious consideration of a robust alpha spending approach. Therefore, the ratio of z=1 is a fair and realistic analysis of the gains from using a robust alpha spending with binary/binomial data.

The total number of events (exposed+unexposed) per hypothesis test was uniformly sampled from the set {1,2,…,10}. This nonconstant sample size per hypothesis test aims to mimic real‐world data arrival frequency, which is unpredictable.

When the total number of adverse events is incorrect because some of the events were not actual events, that is, neither from the treatment/exposed group nor from the control/unexposed group, the actual signaling threshold should be smaller than or equal to that calculated with the wrongly attributed additional events. However, inflation in the type I error probability occurs when the actual number of events in the exposed/treatment group is smaller than that assumed during the sequential test. The error margins selected by the analyst, $E_{n}^{+}$ and $E_{n}^{-}$, must cover the possible positive variations of the difference ($e_{n}^{+}-e_{n}^{-}$) between the wrongly classified events. Therefore, this simulation study evaluates the effects on the Type 1 error and power by over‐ and under‐predicting the number of events that can cumulatively appear during the sequential analysis.

In Table 1, in each of the tabular blocks of type I error probability, statistical power, and expected time to signal (ETS), the diagonal represents scenarios where the counting error margin is perfectly predicted and prespecified by the analyst. Type I error probability presented here, also known as the test size, is the maximum type I error observed under the null hypothesis, that is, r=1. As expected, the type I error is inflated when the actual error margin exceeds the preplanned error margin, that is, $e_{n}^{+}-e_{n}^{-}>E_{n}^{+}-E_{n}^{-}$. On the other hand, if the preplanned error margin exceeds the actual error margin, then there may still be alpha left to be spent at the end of planned surveillance (i.e., overly conservative).

**TABLE 1:** Maximum Type I error probability (test size), statistical power, and expected time to signal (ETS) estimates following the binary/binomial robust alpha spending for selected combinations of counting error margin allowances $E_{n}^{+}-E_{n}^{-}$, with related maximum length of surveillance (N), and actual differences due to wrongly classified events, $e_{n}^{+}-e_{n}^{-}$.

		$E_{n}^{+}-E_{n}^{-}=0$	$E_{n}^{+}-E_{n}^{-}=1$	$E_{n}^{+}-E_{n}^{-}=2$	$E_{n}^{+}-E_{n}^{-}=3$
		N=120	N=120	N=125	N=130
Type I error prob.	$e_{n}^{+}-e_{n}^{-}=$ 0	0.051	0.027	0.017	0.012
	$e_{n}^{+}-e_{n}^{-}=$ 1	0.068	0.049	0.026	0.015
	$e_{n}^{+}-e_{n}^{-}=$ 2	0.144	0.066	0.041	0.023
	$e_{n}^{+}-e_{n}^{-}=$ 3	0.182	0.103	0.065	0.041
Power	$e_{n}^{+}-e_{n}^{-}=$ 0	0.968	0.947	0.945	0.927
	$e_{n}^{+}-e_{n}^{-}=$1	0.977	0.963	0.960	0.939
	$e_{n}^{+}-e_{n}^{-}=$ 2	0.989	0.979	0.971	0.961
	$e_{n}^{+}-e_{n}^{-}=$ 3	0.994	0.986	0.974	0.976
ETS	$e_{n}^{+}-e_{n}^{-}=$ 0	52.84	60.41	69.51	78.14
	$e_{n}^{+}-e_{n}^{-}=$ 1	45.21	54.89	64.51	72.38
	$e_{n}^{+}-e_{n}^{-}=$ 2	37.43	48.93	57.91	67.94
	$e_{n}^{+}-e_{n}^{-}=$ 3	34.54	43.50	55.78	64.51

By construction, even perfect prediction may still leave α available to spend because of the significance level constraint. Similarly, with a minimum requirement, the statistical power observed tends to be greater than the target power even when there are no errors observed, that is, $e_{n}^{+}-e_{n}^{-}=0$ and $E_{n}^{+}-E_{n}^{-}=3$, leading to the closest value (0.927) to the target, 0.9, among the 16 scenarios shown in Table 1. The statistical power observed above the target power is due to the irregular data arrival and departure from a strictly continuous process. The ETS in Table 1 gives the number of events that are required to be observed in both groups (i.e., treatment and reference) and is calculated under the alternative hypothesis (i.e., r=2). More information (in terms of events) is required to generate a signal when expected counting errors are unrealized, that is, $e_{n}^{+}-e_{n}^{-}=0$ and $E_{n}^{+}-E_{n}^{-}=3$, because the total information necessary to end the analysis is artificially longer. On average, 9 additional events are required to accrue to generate a signal when counting error allowances exceed counting errors observed, $E_{n}^{+}-E_{n}^{-}$. When counting errors observed exceed their allowable margins, Type I error is not maintained but also statistical power is artificially inflated with false events and ETS is artificially shorter.

### 3.2 Poisson Data

A baseline rate $\mu_{t}=1$ was used to generate the Poisson counts of events per test. Table 2 shows that the robust alpha spending method produces a conservative type I error when the prespecified error margin exceeds the actual error margin, but it ensures type I error probabilities satisfactorily close to the nominal α level when the predicted error margin reflects the actual error margin. The statistical power pattern is similar to the binomial described above, with all power estimates constrained to be above the target power according to the robustness notion in Definition 2.2. The ETS in the Poisson context is increased by a fraction much smaller than that estimated for the binary/binomial case. As shown in Table 2, the ETS increases by about two units on average for each change in the differential between predicted and actual errors observed for $S_{t}^{+}$. While both the binomial and Poisson are discrete probability distributions, the former has fewer potential solutions since both the exposed and unexposed events are integer‐valued. In the Poisson analysis, only the exposed events must be integers.

**TABLE 2:** Maximum Type I error probability (test size), statistical power, and expected time to signal (ETS) estimates following the Poisson robust alpha spending for selected combinations of counting error margin allowances $S_{t}^{+}$, with related maximum length of surveillance (N), and actual wrongly added events $s_{t}^{+}$.

		$S_{t}^{+}=$0	$S_{t}^{+}=$1	$S_{t}^{+}=$2	$S_{t}^{+}=$3
		N=18.42	N=18.50	N=21.50	N=25.00
Type I error prob.	$s_{t}^{+}=$ 0	0.048	0.032	0.018	0.014
	$s_{t}^{+}=$ 1	0.085	0.047	0.025	0.014
	$s_{t}^{+}=$ 2	0.119	0.077	0.052	0.028
	$s_{t}^{+}=$ 3	0.170	0.128	0.068	0.047
Power	$s_{t}^{+}=$ 0	0.964	0.952	0.932	0.911
	$s_{t}^{+}=$ 1	0.981	0.967	0.954	0.942
	$s_{t}^{+}=$ 2	0.992	0.980	0.978	0.957
	$s_{t}^{+}=$ 3	0.991	0.990	0.978	0.970
ETS	$s_{t}^{+}=$0	16.06	18.49	20.97	23.44
	$s_{t}^{+}=$1	14.13	16.56	19.12	21.04
	$s_{t}^{+}=$2	13.07	14.97	16.57	18.89
	$s_{t}^{+}=$3	12.51	13.46	14.98	16.75

### 3.3 Conditional Poisson Data

The conditional Poisson data were simulated considering historical numbers of events c equal to 10, 30, and 50, which cover the range of possible values used in real applications [22]. The common historical and surveillance person‐times under $H_{0}$ were λ=1. The results indicate that the Type I error is kept under control under the desired significance level as shown in Table 3, and, as by construction, the target statistical power is achieved in each evaluated scenario. The impact on the ETS is moderate, as it increases by about 2 to 3 events per unit increase in error margin, $S_{j}^{+}$.

**TABLE 3:** Maximum Type I error probability (test size), statistical power, and expected time to signal (ETS) estimates following the Conditional Poisson robust alpha spending for selected combinations of counting error margin allowances $S_{j}^{+}$, with related maximum length of surveillance (K), and actual wrongly added events $s_{j}^{+}$, and c=50.

		$S_{j}^{+}$= 0	$S_{j}^{+}$= 1	$S_{j}^{+}$= 2	$S_{j}^{+}$= 3
		K=30	K=33	K=39	K=44
Type I error prob.	$s_{j}^{+}=$ 0	0.054	0.045	0.025	0.021
	$s_{j}^{+}$= 1	0.104	0.050	0.041	0.022
	$s_{j}^{+}$= 2	0.132	0.073	0.051	0.04
	$s_{j}^{+}$= 3	0.179	0.101	0.068	0.046
Power	$s_{j}^{+}$= 0	0.924	0.905	0.903	0.901
	$s_{j}^{+}$= 1	0.938	0.938	0.920	0.916
	$s_{j}^{+}$= 2	0.960	0.953	0.935	0.937
	$s_{j}^{+}$= 3	0.970	0.961	0.958	0.950
ETS	$s_{j}^{+}$= 0	15.47	17.94	20.79	23.55
	$s_{j}^{+}$= 1	15.11	17.08	18.98	22.23
	$s_{j}^{+}$= 2	14.24	16.29	18.96	20.69
	$s_{j}^{+}$= 3	13.78	16.28	18.02	19.90

## 4 Examples

This section utilizes real data and simulated data of adverse events to show how the use of a robust alpha spending works in practice. The calculations were performed with the *Analyze.Binomial* function of the R *Sequential* package, and the programming code to reproduce the calculations is placed in the Appendix A. The signaling threshold was based on an overall alpha level of 0.05.

### 4.1 Real Data

The data used here were extracted from [12]. The data were to support a proof‐of‐concept paper using a known positive adverse event following medical product exposure (i.e., the rejection of $H_{0}$ was expected to occur). Data stability was examined by extracting data from the same time period twice in two different “refreshes.” The initial dataset was extracted closer in time to the data‐lock point, and the subsequent dataset was extracted several months later, when the data were allowed more time to “settle.” In both cases, the data came from nine data sites. Besides the number of events, no other information (e.g., baseline covariate assessment, propensity score estimation and match/strata assignment) changed between downloads.

Table 4 shows the counts of events for the first hypothesis test under the null hypothesis. This table brings the information from both the corrected (i.e., subsequent refresh) and the non‐corrected information (i.e., initial refresh). The incorrect count occurred in the comparison group in the at‐risk population associated with the probability 0.5 (second line of Table 4), where 2 events were registered in the initial refresh, shown in parentheses in the table. Later, in the subsequent refresh of the same time period, one outcome of the comparison group in that same at‐risk population disappeared. Therefore, only one event actually occurred in the comparison group associated with the probability of exposure of 0.5 when the data had been given time to settle.

**TABLE 4:** Information for the first look at the data explored by [12].

Probability of exposure	Treatment group outcomes	Comparison group outcomes	Total outcomes
0.49798	1	0	1
0.50000	0	1 (2)	1 (2)
0.50977	1	0	1
0.50982	1	0	1
0.50983	1	0	1
0.51060	1	0	1
0.51091	0	1	1
0.51169	1	0	1
0.51177	1	0	1
0.1190	1	0	1
0.51198	1	0	1
0.51221	1	0	1
0.51256	1	0	1
0.51345	1	0	1
0.51429	1	0	1
0.51569	0	1	1
0.51582	1	0	1
0.51821	1	0	1
Total	16	3 (4)	19 (20)

*Note:* Non corrected information are in brackets.

Figure 1 shows the main analysis results based on the first hypothesis test using the initial data refresh of Table 4 with the non‐robust (traditional) alpha spending design. The output contains the counts of events, the relative risk estimate, the signaling threshold (in the scale of the events), and the alpha spending. If the data associated with the probability 0.5 was correct, the actual alpha spending would be equal to 0.0077, implied by the signaling threshold of CV=16. Because the observed number of events, 16, reached CV, the conclusion is that there is evidence of an elevated relative risk in the treatment group, and the null hypothesis was rejected.

**FIGURE 1:**
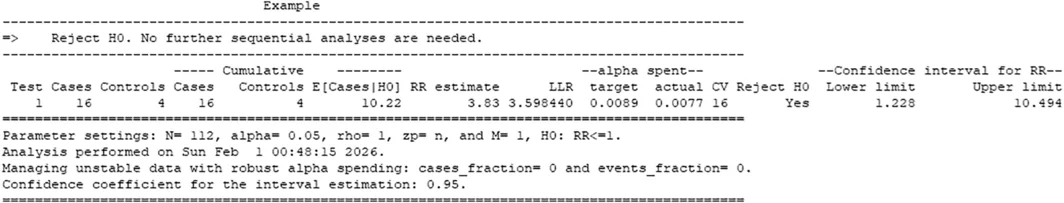
Output table for the first hypothesis test based on the initial (incorrect) data refresh of Table 4 with a traditional alpha spending approach.

The disappearance of outcomes from the comparison group does not inflate the Type I error probability because the effect is to artificially increase the critical value. Larger overall sample sizes lead to larger critical values. For example, if the wrong information was never entered in these data, then the total number of events would be 19 instead of 20. Figure 2 shows the output table after running the first hypothesis test for the corrected data (observable on the subsequent refresh) with the non‐robust alpha spending again. The critical value is the same as before, but the actual alpha spending is slightly smaller, 0.003. Therefore, there is a potential power reduction in the context where outcomes disappear from the comparison group.

**FIGURE 2:**
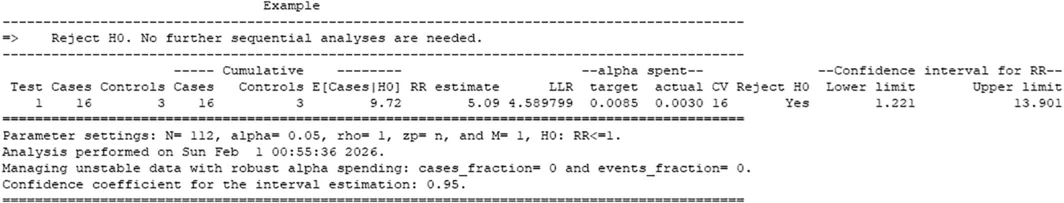
Output table for the first hypothesis test based on the subsequent (corrected) data refresh of Table 4 with a traditional alpha spending approach.

The use of a robust alpha spending implies a small conservatism to avoid incorrectly rejecting the null hypothesis at the beginning of the analysis when the sample size is still small. Figure 3 shows the output table after running the first hypothesis test for the non‐corrected data using the robust alpha spending approach. For this analysis, we parameterized a counting error margin allowance $E_{n}^{+}-E_{n}^{-}=1$. In this case, the critical value is equal to 17, hence the null hypothesis is no longer rejected in the first look at these data. Naturally, since this demonstration proof‐of‐concept was using a known positive when signaling was the expectation, the subsequent analysis would have produced the signal. Notice that the desired statistical power is accommodated by the higher maximum length of surveillance in the robust alpha spending approach (N=117 in Figure 3 compared to N=112 in Figure 2).

**FIGURE 3:**
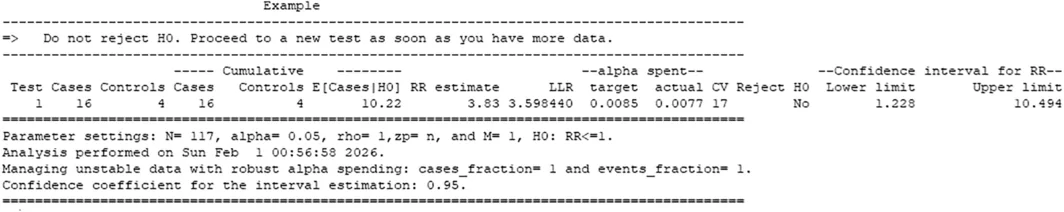
Output table for the first hypothesis test based on the initial data refresh of Table 4 with a robust alpha spending approach.

Now, suppose that the incorrect total of events resulted in a treatment group outcome being removed, leaving a new total of 15 events in the treatment group and 4 events in the comparison group. The robust alpha spending approach is prepared to hold the decision of not rejecting $H_{0}$ in a situation where $e_{n}^{+}-e_{n}^{-}=1$, which in this case leads to CV=17 (Figure 3), then the final decision, by construction, is not directly affected by the wrong information.

### 4.2 Simulated Data

To better illustrate the role of the robust sequential analysis in a realistic way, keeping the same logic of Section 3.1 on mirroring the unpredictability of sample sizes in real applications, consider that the number of binary events (exposed+unexposed) for each hypothesis test l, denoted by $\eta_{l}$ and shown in the second column of Table 5, is uniformly sampled from the set {1,2,…,10}. Then, the number of binomial events among the $\eta_{l}$ events from the exposed group is generated using z=1 and R=1, that is, $p_{i}(1)=1/(1+1/1)=0.5$.

**TABLE 5:** Simulated (R=1 and z=1) example of binary sequential analysis with robust (margin allowances $E_{n}^{+}-E_{n}^{-}=2$, $S_{n}^{+}=0$, $S_{n}^{-}=0$), and non‐robust alpha spending under α=0.05.

l	$\eta_{l}$	n	$c_{l}^{+}$	$c_{l}^{-}$	$e_{n}^{+}$	$e_{n}^{-}$	$e_{n}^{+}-e_{n}^{-}$	$y_{n}$	Robust $cv_{n}$	Non‐robust $cv_{n}$
1	1	1	0	1	0	1	−1	1	NA	NA
2	3	4	1	0	1	1	0	2	NA	NA
3	6	10	0	0	1	1	0	6	12	10
4	10	20	0	0	1	1	0	10	18	16
5	2	22	0	0	1	1	0	10	18	16
6	2	24	0	0	1	1	0	10	21	19
7	2	26	0	0	1	1	0	11	22	20
8	6	32	0	1	1	2	−1	16	26	24
9	8	40	0	0	1	2	−1	20	30	28
10	6	46	0	0	1	2	−1	23	34	32
11	8	54	0	0	1	2	−1	26	38	36
12	4	58	0	0	1	2	−1	27	40	38
13	9	67	0	0	1	2	−1	31	45	43
14	7	74	1	0	2	2	0	34	49	47
15	1	75	0	0	2	2	0	34	49	47
16	10	85	0	1	2	3	−1	38	55	53
17	8	93	0	0	2	3	−1	40	60	57
18	8	101	1	1	3	4	−1	44	64	62
19	4	105	0	0	3	4	−1	47	66	64
20	8	113	0	0	3	4	−1	53	70	68
21	7	120	2	0	5	4	1	56	74	72

For test l, wrongly classified events were drawn without replacement among the $\eta_{l}$ cases with probability 0.1. Then, they were randomly assigned to the two exposure groups with probability 0.5.

For the tuning parameters, the length of surveillance was calculated in order to produce a statistical power of 0.9 for R≥2, which corresponds to N=112 (see the Appendix A—Part A). To reach the desired 0.9 power under the specified error margin, the maximum sample size is automatically adjusted to N=117 by the *AnalyzeSetUp.Binomial* function of the *Sequential* package. For the error margin parameter, we used $E_{n}^{+}-E_{n}^{-}=2$.

For a clear accounting of the data changes at each hypothesis test in the data, define the test‐specific changes $c_{l}^{+}=e_{n}^{+}-e_{n-1}^{+}$ and $c_{l}^{-}=e_{n}^{-}-e_{n-1}^{-}$, fourth and fifth columns of Table 5, with $e_{0}^{+}=e_{0}^{-}=0$. The observed number of events, $y_{n}$, differs from the actual number of events by the cumulative deviation $e_{n}^{+}-e_{n}^{-}$. Note that they “net” deviation of misclassification never exceeded the margin allowance of 2 events even though more than 2 events are overall misclassified. Thus, with the added margin allowance, low levels of misclassification, even if coming in at a constant rate, would not lead to false positive signaling. The last two columns of Table 5 show the critical values of the robust and the non‐robust alpha spending plans, respectively. It is clear that, when the tuning parameters $S_{n}^{+}$ and $S_{n}^{-}$ are fixed to zero (i.e., there are no “lost” overall events or late‐arriving events), the critical value curve is simply increased by $E_{n}^{+}-E_{n}^{-}$ units. Different signaling threshold formats (i.e., different scales to measure the test statistic) may appear with more sophisticated choices for the tuning parameters $E_{n}^{+}$, $E_{n}^{-}$, $S_{n}^{+}$, and $S_{n}^{-}$.

## 5 Concluding Remarks

The violations of the anchoring assumption in sequential hypothesis testing are expected to be controlled with the method shown here. The most important feature of this procedure is that it does not require the user to make adjustments to the data during the sequential analysis if and when incorrectly attributed events have been used in previous tests. In fact, the method was designed to be robust to potentially inconsistent data even when they are never detected by the analyst.

To operationalize the proposed robust alpha spending, the user must predefine the maximum amount of counting errors that can occur during the sequential analysis. These values should be based on historical performance where possible, or quantification using a unit test approach. That is, for data sources that are continually updated (e.g., weekly, monthly), it should be easy to repeatedly generate a test dataset (sometimes called a “unit test”) each time data are refreshed and quantify changes in the dataset over time. With a 10% error rate in the total data, a margin allowance of 2 events appears sufficient to deal with that net level of change. Data‐driven and empirical ways to estimate the margin allowances will be explored in future investigations.

In general, any adaptation to account for greater uncertainty or variance in an estimate is going to result in a statistical power penalty, which may be realized in a delayed time‐to‐signal or a longer surveillance period before one fails to reject the null hypothesis. It is like any form of “insurance” that might later be shown to be unnecessary. Still, in many cases, this additional waiting time may be well worth the price of greater confidence in the signaling decision. With rare events that occur once in every 100,000 persons, perhaps waiting for 2 additional events may carry a greater penalty in study resources and time. Further, in cases where the matching ratio is less than 1 (i.e., the risk window or group is expected to be much larger than the comparison window), such a correction may be less relevant and worthwhile.

## Funding

This work was supported by the Conselho Nacional de Desenvolvimento Científico e Tecnológico (Grant No. 302882/2022‐7), Fundação de Amparo à Pesquisa do Estado de Minas Gerais (Grant Nos. PQ‐00787‐21 and RED‐00133‐21), and the Robert H. Ebert Career Development Award (Finance Code 001).

## Conflicts of Interest

The authors declare no conflicts of interest.

## Data Availability

The data that supports the findings of this study is available in the body of this article.

## Appendix A

This appendix brings the command lines to run the examples in Section 4 using the R *Sequential* package.

### Part A‐ Code to Calculate the Maximum Length of Surveillance

The minimum sample size, or maximum length of surveillance (N), needed to ensure a statistical power of 0.9 for R=2 run with the following command:
which returned N=112.

### Part B‐ Code to Run the Analysis Results of Figure 1

The parameterization of the sequential analysis hyperparameters was run with the following command:
The code to run the first test and obtain the output table shown in Figure 1 was run with the following command:

### Part C‐ Code to Run the Analysis Results of Figure 2

The parameterization of the sequential analysis hyperparameters was run with the following command:
The code to run the first test and obtain the output table shown in Figure 2 was run with the following command:

### Part D‐ Code to Run the Analysis Results of Figure 3

The parameterization of the sequential analysis hyperparameters was run with the following command:
The code to run the first test and obtain the output table shown in Figure 3 was run with the following command:

## References

[1] S. Gandhi , M. Iannacone , A. Leapley , et al., “Real‐Time Pharmacovigilance: Transforming Population‐Based Monitoring of Post‐Approval Vaccine Safety Through Rapid Cycle Analysis (RCA)‐A Review of the Published Literature,” Pharmaceuticals 18, no. 1 (2025): 80, 10.3390/ph18010080. PMC11769534 39861142

[2] A. Leite , N. Andrews , and S. Thomas , “Near Real‐Time Vaccine Safety Surveillance Using Electronic Health Records: A Systematic Review of the Application of Statistical Methods,” Pharmacoepidemiology and Drug Safety 25 (2016): 225–237. 10.1002/pds.3966 PMC5021108 26817940

[3] W. Yih , M. Kulldorff , B. Fireman , et al., “Active Surveillance for Adverse Events: The Experience of the Vaccine Safety Datalink Project,” Pediatrics 127, no. Suppl. 1 (2011): S54–S64. 10.1542/peds.2010-1722I 21502252

[4] T. Lieu , M. Kulldorff , R. Davis , et al., “Real‐Time Vaccine Safety Surveillance for the Early Detection of Adverse Events,” Medical Care 45, no. S (2007): 89–95. 10.1097/MLR.0b013e3180616c0a 17909389

[5] R. Davis , M. Kolczak , E. Lewis , et al., “Active Surveillance of Vaccine Safety: A System to Detect Early Signs of Adverse Events,” Epidemiology 16 (2005): 336–341. 10.1097/01.ede.0000155506.05636.a4 15824549

[6] K. K. G. Lan and D. L. DeMets , “Discrete Sequential Boundaries for Clinical Trials,” Biometrika 70, no. 3 (1983): 659–663.

[7] C. Jennison and B. W. Turnbull , “Statistical Approaches to Interim Monitoring of Medical Trials: A Review and Commentary,” Statistical Science 5 (1990): 299–317.

[8] S. Pocock , “Group Sequential Methods in the Design and Analysis of Clinical Trials,” Biometrika 64 (1977): 191–199.

[9] P. O'Brien and T. Fleming , “A Multiple Testing Procedure for Clinical Trials,” Biometrics 35 (1979): 549–556. 497341

[10] S. K. Wang and A. A. Tsiatis , “Approximately Optimal One‐Parameter Boundaries for Group Sequential Trails,” Biometrics 43 (1987): 193–200. 3567304

[11] M. Kulldorff , R. L. Davis , M. Kolczak , E. Lewis , T. Lieu , and R. Platt , “A Maximized Sequential Probability Ratio Test for Drug and Vaccine Safety Surveillance,” Sequential Analysis 30 (2011): 58–78.

[12] J. Maro , E. Eworuke , L. Hou , et al., “Conducting Prospective Sequential Surveillance in Real‐World Dynamic Distributed Databases,” Pharmacoepidemiology and Drug Safety 20, no. 10 (2020): 1331–1335. 10.1002/pds.5002 32449261

[13] I. Silva , J. Maro , and M. Kulldorff , “Exact Sequential Test for Clinical Trials and Post‐Market Drug and Vaccine Safety Surveillance With Poisson and Binary Data,” Statistics in Medicine 40, no. 22 (2021): 4890–4913. 10.1002/sim.9094 PMC8441767 34120357

[14] J. Nelson , R. Wellman , O. Yu , et al., “A Synthesis of Current Surveillance Planning Methods for the Sequential Monitoring of Drug and Vaccine Adverse Effects Using Electronic Health Care Data,” EGEMS (Washington, DC) 4, no. 1 (2016): 1219, 10.13063/2327-9214.1219. PMC5051582 27713904

[15] L. Li and M. Kulldorff , “A Conditional Maximized Sequential Probability Ratio Test for Pharmacovigilance,” Statistics in Medicine 29 (2010): 284–295. 10.1002/sim.3780 19941282

[16] A. Michnick , S. C. MacDonald , S. Adimadhyam , et al., “Sequential Safety Surveillance of RSVpreF Vaccination During Pregnancy Early in the Postapproval Period,” JAMA Network Open 9, no. 4 (2026): e266190, 10.1001/jamanetworkopen.2026.6190. PMC13100873 42012833

[17] S. Perez‐Vilar , M. Hu , E. Weintraub , et al., “Guillain‐Barre Syndrome After High‐Dose Influenza Vaccine Administration in the United States, 2018–2019 Eason,” Journal of Infectious Diseases 223 (2021): 416–425, 10.1093/infdis/jiaa543. PMC7882024 33137184

[18] J. Donahue , B. Kieke , E. Lewis , et al., “Near Real‐Time Surveillance to Assess the Safety of the 9‐Valent Human Papillomavirus Vaccine,” Pediatrics 144, no. 6 (2019): e20191808, 10.1542/peds.2019-1808. PMC7780202 31740498

[19] I. Silva and M. Kulldorff , “Sequential: Exact Sequential Analysis for Poisson and Binomial Data. R Foundation for Statistical Computing‐ Contributed Packages; Vienna, Austria,” R Package version 4.3.3, (2024).

[20] I. Silva and M. Kulldorff , “Continuous Versus Group Sequential Analysis for Post‐Market Drug and Vaccine Safety Surveillance,” Biometrics 71, no. 3 (2015): 851–858. 10.1111/biom.12324 PMC4888780 26011024

[21] I. Silva , “Type I Error Probability Spending for Post‐Market Drug and Vaccine Safety Surveillance With Poisson Data,” Methodology and Computing in Applied Probability 20, no. 2 (2017): 739–750. 10.1007/s11009-017-9586-z PMC6936745 31889890

[22] I. Silva , W. Lopes , P. Dias , and K. Yih , “Alpha Spending for Historical Versus Surveillance Poisson Data With CMaxSPRT,” Statistics in Medicine 38, no. 12 (2019): 2126–2138. 10.1002/sim.8097 PMC6955154 30689224

